# Botulinum Neurotoxin Therapy in the Clinical Management of Laryngeal Dystonia

**DOI:** 10.3390/toxins14120844

**Published:** 2022-12-01

**Authors:** Winnie Yeung, Amanda L. Richards, Daniel Novakovic

**Affiliations:** 1 Voice Research Laboratory, Faculty of Medicine and Health, University of Sydney, Camperdown, NSW 2050, Australia; 2 Department of Otolaryngology, The Canterbury Hospital, Campsie, NSW 2194, Australia; 3 Department of Otolaryngology, The Royal Melbourne Hospital, Parkville, VIC 3050, Australia

**Keywords:** botulinum neurotoxin, laryngeal dystonia, spasmodic dysphonia, injection, electromyography

## Abstract

Laryngeal dystonia (LD), or spasmodic dysphonia (SD), is a chronic, task-specific, focal movement disorder affecting the larynx. It interferes primarily with the essential functions of phonation and speech. LD affects patients’ ability to communicate effectively and significantly diminishes their quality of life. Botulinum neurotoxin was first used as a therapeutic agent in the treatment of LD four decades ago and remains the standard of care for the treatment of LD. This article provides an overview of the clinical application of botulinum neurotoxin in the management of LD, focusing on the classification for this disorder, its pathophysiology, clinical assessment and diagnosis, the role of laryngeal electromyography and a summary of therapeutic injection techniques, including a comprehensive description of various procedural approaches, recommendations for injection sites and dosage considerations.

## 1. Introduction

First described by Traube in 1871 [1], laryngeal dystonia (LD) is a chronic, task-specific, focal movement disorder primarily affecting the essential function of voice production [2,3]. Historically, the terms spasmodic dysphonia (SD) and LD have been used interchangeably [4]. The latter, LD, is now widely adopted due to updated nomenclature [4]. LD is a rare neurological condition with a prevalence of 1–6 per 100,000 population [5,6,7]. It has a female preponderance [4] with an overall ratio of 4:1 [8]. Patients typically present in middle age, between the 4th and 6th decade of life [7,9,10].

Whilst the aetiology remains unclear, many environmental factors are implicated in LD [4,8,11]. Schweinfurth et al. found that 30% of patients associated the onset of symptoms with an upper respiratory tract infection, while 65% of LD patients have previously had measles or mumps, compared to a national average of 15%. [12]. Another 21% of patients correlated their initial presentation with a stressful major life event [12]. In addition to viral insults and emotional stressors, gastroesophageal reflux and neck trauma have been identified as potential triggers for the manifestation of LD symptoms in susceptible individuals [13]. While 12% of LD patients have a family history of dystonia, a specific gene for LD has not been identified [2,11].

The injection of botulinum neurotoxin into the intrinsic laryngeal muscles to treat LD was pioneered by Brin and Blitzer in 1984 [14]. The efficacy of Botulinum Neurotoxin Type A (BoNT-A) chemodenervation treatment has since been supported by a large body of evidence [2,3,9,14,15,16]. It continues to be the standard of care for LD today.

### 1.1. Pathophysiology

The neuroanatomy and neurophysiology of phonation are complex. The first report suggesting a neurological origin for LD demonstrated aberrations in the temporal region of LD patients on electroencephalography in 1960 [17]. Although the exact aetiology of LD remains poorly understood, knowledge has evolved at pace in the past decade. Current evidence suggests that there is both a structural and functional component to the pathophysiology of LD.

Recent studies have revealed LD to be a somatosensory disorder associated with structural alterations in brain organisation [18]. Multiple abnormal structural changes in both white and grey matter have been identified in focal dystonias [19]. Analysis of phenotypes and genotype-specific structural differences of LD patients using high-resolution MRI and diffusion-weighted imaging showed that evaluating structural abnormalities alone was sufficient in differentiating between different subtypes of LD [19]. Furthermore, abnormal functional connectivity within the sensorimotor and frontoparietal networks was seen in LD patients [20]. These changes allow the differentiation of LD patients from normal subjects with 71% accuracy [8,21].

Loss of cortical inhibition appears to be another feature in both motor and sensory systems of dystonia patients. Measurement of the cortical silent period (CSP) using transcranial magnetic stimulation found decreased CSP in the masseter and first interosseus muscles in LD patients, compared to healthy controls [22]. Shortened CSP in phenotypically unaffected muscles suggests reduced cortical inhibition [23].

Somatosensory disturbances are seen in both the central and peripheral nervous systems. A positive correlation between LD severity and increased activation intensity in the left somatosensory cortex in functional MRI studies [24]. Irregularities in tactile and visual temporal discriminations have been associated with focal dystonia [25,26]. LD patients exhibited impaired limb proprioception [27], suggesting that peripheral proprioceptive dysfunction is global rather than restricted to the area affected by the focal dystonia alone [27,28,29,30].

### 1.2. Mechanism of Action of Botulinum Toxin in LD

Botulinum neurotoxin (BoNT) is a potent toxin synthesised by the clostridial species [31,32]. The biologically active 150-kd core neurotoxin protects itself from thermal damage, pH stresses and enzymatic degradation by forming a stable molecular complex with other non-toxin proteins [33]. Several immunologically distinct serotypes of BoNT have been discovered, named A through G [33,34]. Types A and B cause disease in humans but have also been harnessed for commercial and medical use [35,36]. Its mechanism of action involves the reversible disruption of the exocytotic process within neurons at localised and highly specific sites, preventing the release of the neurotransmitter acetylcholine (Ach) from axon endings at the neuromuscular junctions (NMJ) [37]. Following injection into specific intrinsic laryngeal muscles, BoNT leads to flaccid paralysis, forming the basis for functional improvement in LD through chemodenervation [38,39,40].

Although it is widely accepted that the primary therapeutic actions of BoNT are related to the peripheral nervous system, there is evidence that BoNT may exert effects beyond the locally treated NMJ [41,42]. These additional actions of BoNT at distant sites have been supported by a number of neurophysiological [43,44,45] and neuroimaging studies [46,47,48], as well as clinical observations [49,50,51]. Unilateral BoNT injections into laryngeal muscles reduce muscle activity in both the treated and contralateral untreated side in LD, suggesting an effect upon central pathophysiology [52]. The exact mechanisms of this effect remain unclear, but alterations of the sensory feedback mechanism along with central effects due to retrograde transport of botulinum toxin have been postulated [53]. More recently, functional MRI studies have shown reduced activity in specific brain regions (left precuneus region) in LD patients successfully treated with BoNT compared with unsuccessfully treated and untreated patients. Moreover, subtype-specific regions of decreased activity were noted for the adductor (ADLD) (right thalamus) and abductor (ABLD) (left inferior frontal cortex) variants respectively [54]. Studies evaluating symptomatic improvement with BoNT treatment or peripheral stimulation show direct effects on muscle spindle as well as normalisation of cortical sensory organisation and function [43,47,55], implying correction of proprioceptive dysfunction as a mechanism for therapeutic response.

## 2. Clinical Presentation of Laryngeal Dystonia

### 2.1. Classification of Laryngeal Dystonia

**Figure 1:**
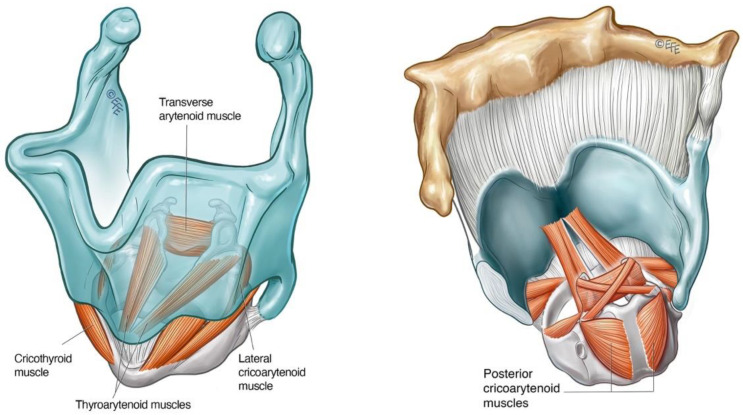
Schematic diagram of the larynx depicting the intrinsic laryngeal muscles, highlighting the thyroarytenoid (TA) and lateral crico-arytenoid (LCA) from the adductor muscle group (**left**) and the posterior crico-arytenoid (PCA) abductor muscle (**right**).

LD is classified according to the primary intrinsic laryngeal muscle groups affected (Figure 1, Table 1). Adductor laryngeal dystonia (ADLD) is the most common form of LD, comprising over 80% of cases [9]. ADLD is characterised by the intermittent abnormal activity of the thyroarytenoid (TA)/lateral cricoarytenoid (LCA) adductor muscle complex, resulting in involuntary glottic closure or squeeze/spasms/strain during phonation. Abductor spasmodic dysphonia (ABLD) accounts for 10–15% of focal laryngeal dystonia [2,56]. It is characterised by the abnormal involuntary activity of the posterior cricoarytenoid (PCA) muscle during phonation, giving rise to breathy and asthenic voice quality. A hybrid variant, mixed laryngeal dystonia (Mixed LD), in which patients exhibit clinical features of both ADLD and ABLD [2,57,58], is rare, accounting for <5% of cases [59], and can be more challenging to treat in comparison to other subtypes of LD. Other rarer variants of LD, affecting specific tasks of breathing [2] and singing [60], have also been described.

### 2.2. Clinical Assessment and Diagnosis

The diagnosis of laryngeal dystonia relies on detailed patient clinical history, along with perceptual voice analysis and recognition of vocal spasm patterns characteristic of each subtype [2,65,66]. Symptoms are ameliorated by alcohol in up to 58% of patients [8,67,68]. Sensory tricks (‘Geste antagoniste’), such as the physical gesture of touching the ear, for example, may temporarily disrupt the dystonia and cause the voice to improve [4,8,69]. Approximately 25–30% of patients with LD will exhibit a focal action-induced tremor [70,71]. The patient is invited to read examples of voice-weighted [61,72,73] and voiceless-weighted [56] phrases aloud. (Table 2 and Table 3). These phrases are specifically designed to unmask the adductor and abductor subtypes of LD, respectively [56,61,73]. The focus of comprehensive head and neck and generalised physical examination is directed at eliciting tremors or abnormal muscle activity in other parts of the body, including facial twitching or spasms.

An endoscopic laryngeal examination is essential to exclude structural or mucosal lesions, as well as eliciting signs of peripheral neurological weakness [74]. One must be aware that the endoscope itself may unwittingly serve as a ‘geste antagoniste’, yielding an apparently normal examination. Characteristic laryngoscopic patterns of abnormal adductor activity are best elicited by asking the patient to count with voice-weighted numbers (in English, from eighty to ninety) [4]. Abnormal abductor activity is typically discerned when patients count with voiceless-weighted numbers (in English from sixty to seventy) [4]. Supraglottal patterns of muscle tension constriction (which may be compensatory in ABLD) on sustained phonation and connected speech, may be observed [2,75,76]. These findings may help guide treatment.

Consensus-based attributes have been developed as a guideline to help clinicians classify patients with ADLD and ABLD and distinguish them from other conditions with similar or overlapping voice symptoms [61,77].

### 2.3. The Role of Laryngeal Electromyography (LEMG)

Fine-wire EMG studies have been performed on patients with laryngeal dystonia by Hillel [62] and Klotz et al. [78]. These studies demonstrated abnormal EMG activity in all intrinsic laryngeal muscles of patients with LD, with prolonged latencies before phonation onset and increased frequency of post-phonatory activity. Similar EMG findings between the abductor and adductor subtypes of the condition indicate that any of the intrinsic laryngeal muscles can be involved in both subtypes.

A systemic review [79] concluded that whilst there is a clear benefit to LEMG-guided injections into the TA in ADLD, there was no evidence for a difference in accuracy between LEMG and endoscopically guided injection into the PCA in ABLD. The characteristics of motor unit potentials (MUPs), recruitment potentials and laryngeal nerve evoked potentials (EPs) in a cohort of ADLD patients before and after BoNT-A treatment has also been studied [80]. Significantly increased amplitudes of MUPs in the TA muscles were noted on LEMG before treatment. Following BoNT-A injections, in addition to denervation changes on LEMG, EPs were weakened or disappeared in the injected muscle [80].

In view of the current evidence, the authors strongly recommend the use of LEMG to ensure accurate localisation of target muscles during BoNT injection. EMG studies may be considered in those with LD recalcitrant to standard management to identify other target muscles for treatment.

A single-channel EMG machine with acoustic and preferably also visual feedback is adequate for a basic setup. This should be attached to a separate electrical circuit to minimise electrical interference during the procedure. Ground and negative leads are connected to the EMG machine and attached to the patient transcutaneously over the clavicle and sternocleidomastoid muscle, respectively. The authors perform injections using 1.5-inch, hollow-bore 26 or 27-gauge, insulated Teflon or plastic-coated monopolar EMG needles.

## 3. Clinical Application of Botulinum Toxin in Laryngeal Dystonia

Various botulinum toxin preparations are currently available on the commercial market. These include onabotulinium toxin Type A (Botox^®^, Allergan, Irvine, CA, USA), abobotulinum toxin Type A (Dysport^®^, Ipsen, Slough, UK) and incobotulinum toxin Type A (Xeomin^®^, Merz Pharma, Frankfurt am Main, Germany). Whilst most of the LD literature relates to treatment with onabotulinumtoxinA, there is evidence for the successful use of incobotulinumtoxinA [81] and abobutulinumtoxinA [82] in the treatment of LD. Additionally, preparation of botulinum toxin Type B, rimabotulinumtoxinB (Myobloc^®^, Solstice Neurosciences LLC, Rockville, MD, USA) has been used as a safe and effective treatment option for laryngeal dystonia [83,84,85]. Each of these preparations has a unique formulation and varying manufacturing processes, giving rise to different pharmacological properties and requiring different dosing regimens. For consistency and ease of use, all BoNT-A dosing regimens outlined in this article refer to units of onabotulinum toxin Type A (Botox^®^) based on the authors’ clinical experience and the current literature.

To minimise variability associated with volume diffusion of botulinum toxin beyond the intrinsic laryngeal muscles, the authors recommend the use of a consistent volume of 0.1 mL in each vocal fold for ADLD whilst the concentration is adjusted so that the desired units of BoNT-A is delivered. This is achieved by making up a standard stock of 100 units of Botox^®^ with 4 mL of sterile 0.9% NaCl (2.5 units of Botox^®^ per 0.1 mL). The desired concentration of BoNT-A is achieved by further diluting this standard stock (2.5 units per 0.1 mL) with sterile 0.9% NaCl. A 0.1 mL aliquot containing the required units of BoNT-A is then drawn up into a 1-millilitre luer-slip syringe for injection. If higher concentrations of BoNT-A are required, i.e., greater than 2.5 units per 0.1 mL, then a ‘double-strength’ stock can be made with 100 units of Botox^®^ in 2 mL of sterile 0.2% NaCl achieving a concentration of 5 U per 0.1 mL. This can be further diluted to the desire concentration as described above.

### 3.1. Adductor Spasmodic Dysphonia (ADLD)

Patients with ADLD typically present with a strained, tight, and strangled voice, with limited variation in pitch. Harsh, intermittent adductor pitch breaks are most evident in voice-weighted (vowel) sentences (Table 2). Vocal fatigue and difficulties with both volume and projection are typical complaints among this patient group [9,15].

#### 3.1.1. Cricothyroid Membrane Approach to Adductor Muscle Complex

**Figure 2:**
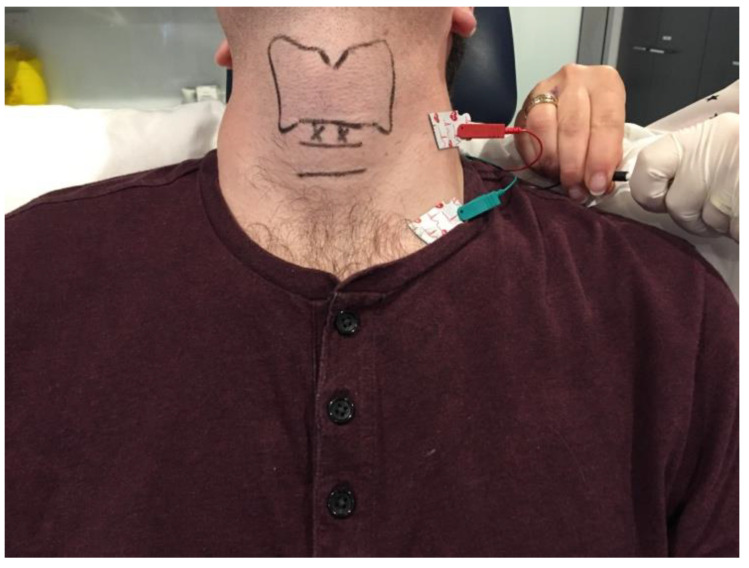
Clinical photograph depicting the ground and negative transcutaneous leads required for laryngeal EMG setup and the landmarks for the cricothyroid membrane approach to the adductor muscle complex. The surface landmarks for the thyroid and cricoid cartilages are delineated in black. The entry points for the injection needle are marked by ‘X’.

**Figure 3:**
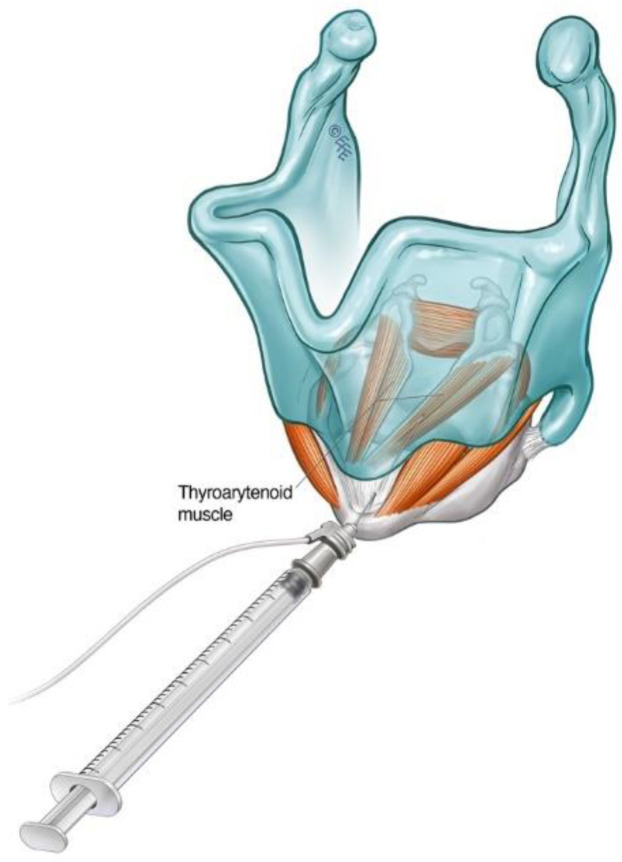
Schematic diagram illustrating the cricothyroid membrane injection approach to TA muscle in the treatment of ADLD.

The patient can be positioned upright or semi-reclined in a treatment chair or supine on an examination couch with the neck slightly extended. The cricothyroid membrane is identified using the superior border of the cricoid cartilage and the inferior border of the thyroid cartilage as landmarks (Figure 2). The needle is inserted via the cricothyroid space approximately 3 mm lateral to the midline and directed 30–45 degrees superolateral (Figure 3). The patient is advised to refrain from coughing, swallowing or phonation unless instructed to do so. The EMG machine is activated, and the needle is advanced into the paraglottic space below the inferior lip of the thyroid cartilage. Sharp, crisp EMG potentials indicate that the motor endplates of the target TA muscle are near the tip of the injection needle. The patient is asked to phonate gently in modal voice for EMG confirmation of needle position before delivery of BoNT. In cases where the LCA muscle is targeted, a similar approach is deployed with the needle angled more posterolateral.

#### 3.1.2. Transthyrohyoid Approaches to the Larynx

**Figure 4:**
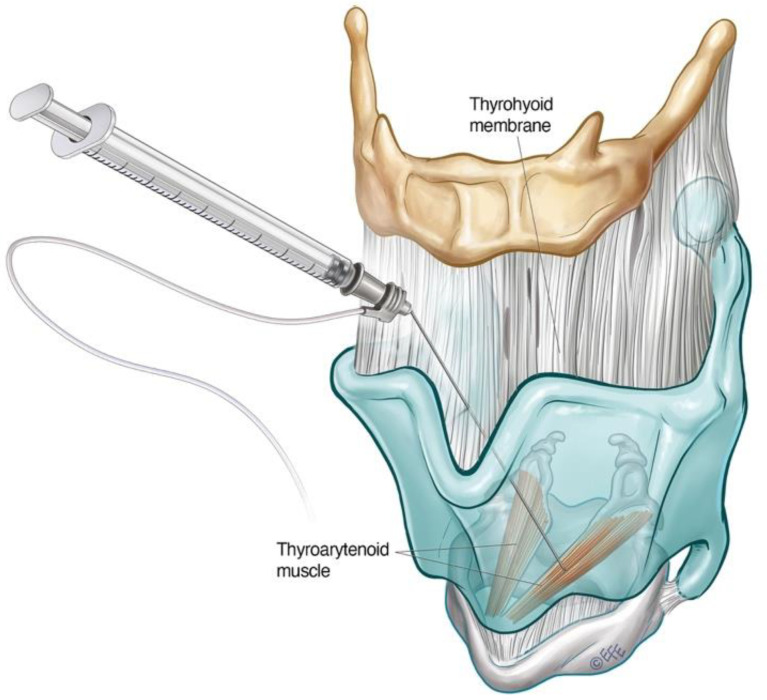
Schematic diagram illustrating transthyrohyoid injection approach to the TA. The supraglottic structures can also be approached in this way.

Where EMG is not available, the adductor complex can alternatively be accessed via a transthyrohyoid approach using flexible transnasal endoscopic guidance according to the method described by Amin [86], where the needle enters the airway and is directed towards the paraglottic space under endoscopic visualisation (Figure 4).

Schönweiler et al. first described a transoral approach for injection of BoNT-A into the supraglottis for treatment of ADLD based on the observation that some ADLD patients exhibited hyperfunction of the supraglottic musculature in addition to the typical glottic ‘squeeze’ [87]. Young and Blitzer subsequently reported good symptom control delivering BoNT-A into the supraglottis via a trans thyrohyoid technique in this subgroup of ADLD patients, who derived limited functional benefit treatment of the TA alone. [75]. Transient mild to moderate dysphagia was experienced in 50% of their small cohort of patients treated in this way [75].

The trans thyrohyoid approach to the supraglottic structures requires endoscopic guidance to ensure accurate toxin delivery. The patient is positioned upright with the head in extension. The thyrohyoid membrane is identified using the superior thyroid notch as a landmark. The needle is inserted through the thyrohyoid space at the midline just above the superior thyroid notch, with a 45-degrees bend at the hub directed inferiorly. Once over the notch, the needle is slowly advanced downwards at an acute angle and rotated slightly towards the side being injected. The position of the needle is confirmed when tenting of the supraglottic mucosa is seen on endoscopy. The toxin can then be delivered submucosally under visualisation

The change of primary injection site from the TA to the supraglottis may offer the advantage of more gradual onset with less severe side-effects of breathiness [76]. In a longitudinal functional study of ADLD patients, 7.5 units (average dose) were delivered into the submucosal space of each false vocal fold. Of the patients, 76% treated in this way reported no decline in the percentage of normal vocal function, whilst 24% experienced only a small transient post-injection decline [76]. In view of these findings, the authors have advocated this approach in the treatment of professional voice users and patients who experience excessive breathiness with TA/LCA injections to minimise vocal downtime.

#### 3.1.3. Interarytenoid BoNT-A Injections for ADLD

Involvement of the interarytenoid (IA) muscle has been demonstrated in fine-wire EMG studies in patients with ADLD [88]. In patients who have not achieved a good therapeutic response with BoNT injections to the TA/LCA muscle complex alone, additional injections to the IA (mean BoNT-A dose 2.0 units) have achieved success in symptom control [88,89]. The needle enters the CT membrane in the midline, directed 30 degrees supero-posteriorly. It is then advanced until the air microphonic on EMG ceases, and mild resistance is re-encountered. The patient is asked to phonate on an ‘eee’ at fundamental frequency until EMG confirmatory signal is heard. Encountering firm resistance without a positive EMG signal indicates the needle has hit the posterior plate of the cricoid cartilage. The needle should be drawn back into the airway and inched superiorly incrementally until the IA muscle is encountered beyond the superior border of the cricoid cartilage.

#### 3.1.4. Dosing and Laterality Considerations in ADLD

The aim of laryngeal BoNT-A treatment for ADLD should be to achieve a fluent voice with improved vocal function for as long as possible whilst minimising the side-effects throughout the treatment cycle [90]. In a 10-year follow-up of over 200 patients, Lerner et al. reported a difference in BoNT-A dose based on gender [91]. The average doses of BoNT-A received by male and female ADLD patients were 0.6 units and 1.3 units on each side, respectively. In contrast, no significant difference in dosage based on age or gender was observed in another large series of 155 patients [92].

In the authors’ practice, 1.0 unit to each vocal fold bilaterally is typically offered at the initial treatment session, as recommended by Blitzer et al. [2]. This may be reduced to 0.6–0.8 units per vocal fold if the vocal function is critical to the patient in the coming weeks. The patient is then reviewed at 2–4 weeks to assess response. Symmetrical vocal fold movement on laryngoscopy with ongoing adductor pitch breaks may indicate under-dosing. A ‘top-up’ dose can be administered bilaterally at this stage.

The average duration of benefit from one treatment cycle is between 11–15 weeks [2,93]. Transient breathiness of the voice is common after ADLD treatment and has been proposed as a marker for successful injection [66,94,95]. Novakovic et al. found that 28.5% of patients experience an initial deterioration in vocal function associated with a weak and breathy voice lasting an average of 20 days (median 14 days) [90]. This initial side effect may be unacceptable to a proportion of patients. Staggered bilateral dosing can be considered to mitigate this [2,96]. One vocal fold is injected, with the patient returning 2–3 weeks after for BoNT-A injection to the contralateral vocal fold. The authors find that some patients prefer to present at regular, closely spaced intervals, on average 6 weeks apart, to receive smaller alternating doses to minimise breathiness and vocal downtime. Unilateral botulinum toxin injections have also been reported as an effective primary treatment for ADLD. Lee et al. [97] have observed that both alternating unilateral and bilateral injection regimes demonstrated comparable levels of efficacy, durability, and stability for the treatment of ADLD. The group concluded that alternating unilateral injections could be routinely performed at shorter intervals, with fewer side-effects compared to bilateral injections. These findings are in contrast to another cohort study [98], which found bilateral BoNT-A injections to be more effective in producing optimal therapeutic effects to side-effect profiles.

### 3.2. Abductor Spasmodic Dysphonia (ABLD)

Patients with abductor spasmodic dysphonia present with a weak and breathy voice with characteristic abductor pitch breaks on connected speech [16,56,61]. These typically manifest as sudden aphonic whispered moments of speech, indicating inappropriate PCA muscle activity [16,56] which are best assessed on voiceless weighted sentences (Table 3). Treatment of ABLD is more challenging than ADLD. The average self-reported best voice achieved is 70% of normal function in this group of patients [2,96]. The maximal dose effect may be limited by the potential for airway compromise. Additional treatment options, including BoNT-A injection to the cricothyroid muscle and vocal fold medialisation [2,99], may be considered when the limit of airway compromise has been reached.

Access to the PCA muscle is more challenging than to the adductor complex. It lies posterior to the larynx, emerging as a broad fan from the posterior surface of the cricoid lamina, coursing obliquely upwards in a lateral direction before inserting into the muscular process of the arytenoid.

#### 3.2.1. Lateral Rotation Approach to PCA Muscle

**Figure 5:**
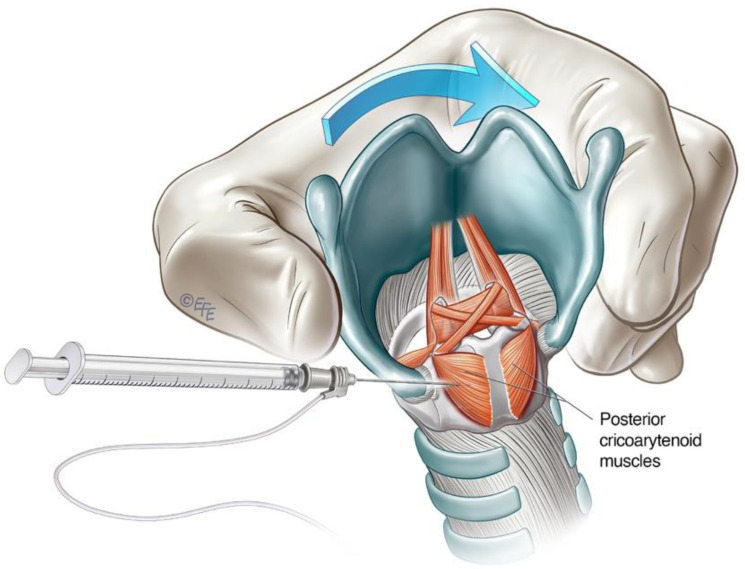
Schematic diagram illustrating lateral rotational injection approach to the PCA.

The lateral rotation injection approach to the PCA is a commonly used technique for ABLD. It allows transcutaneous access to the PCA muscle without breach of the airway [40]. The cricoid cartilage and lateral posterior border of the thyroid cartilage serve as landmarks. The larynx is rotated between the thumb and forefingers such that the thyroid notch is displaced away from the side being injected, thereby opening the posterior face of the cricoid of the injected side for access [16,40] (Figure 5). The needle is inserted along the lower half of and posterior to the thyroid cartilage with care until the posterior plate of the cricoid is encountered. The needle is withdrawn slightly, and the patient is asked to sniff in through the nose to activate the PCA muscle. This is deployed as a confirmatory manoeuvre on LEMG before the toxin is injected. The contralateral side can be treated in a similar way, but one must be aware of the potential for airway compromise in synchronous injections [16,100].

#### 3.2.2. Anterior Trans-Airway Approach to PCA Muscle

Alternative transmucosal access to the PCA muscle via an anterior approach was reported by Rontal et al. [101]. This approach relied on the diffusion of high doses of BoNT-A by injection superior to the cricoid lamina above the PCA muscle under endoscopic guidance. The technique has been modified over time, evolving into a trans-cricoid approach as described by Meleca et al. [102], allowing for more accurate PCA muscle localisation with the aid of EMG.

**Figure 6:**
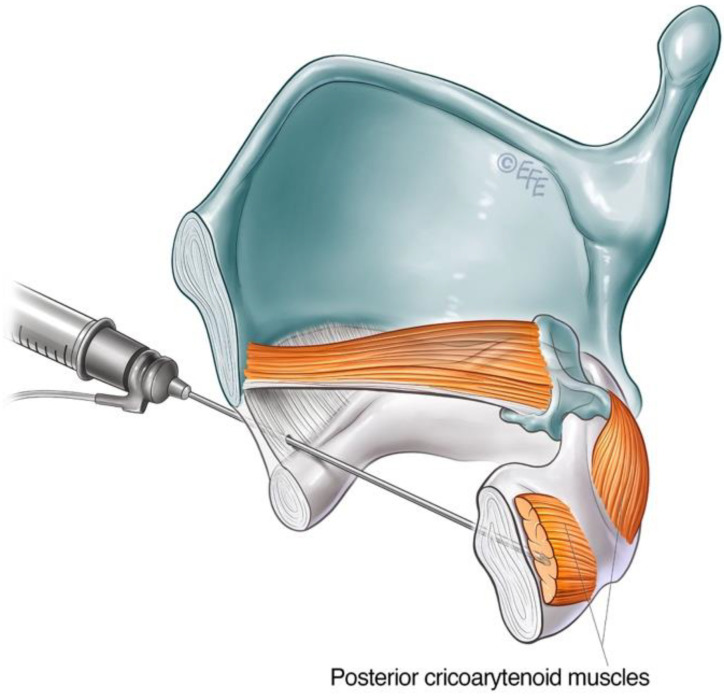
Schematic diagram illustrating anterior trans airway approach to the PCA muscle.

The airway is anaesthetised with a transmucosal injection of 2 mL of lidocaine 2% delivered through the cricothyroid membrane using a 25-gauge needle. This will trigger airway reflexes. The patient is encouraged to cough to distribute the anaesthetic throughout the laryngeal region. The cricoid cartilage and inferior anterior border of the thyroid cartilage are identified as landmarks. The needle is inserted via the cricothyroid space in the midline and directed posteriorly, approximately 30 degrees laterally, towards the target PCA muscle (Figure 6). This approach transverses the cricoid plate. A larger 26-gauge needle may be helpful, especially if calcification of the cartilage has occurred. The patient should refrain from coughing, swallowing or phonate whilst the needle is inserted. The EMG machine is activated as the needle is advanced through the cricothyroid membrane towards the posterior cricoid plate. The injector looks and listens for the airway microphonic (a loud buzzing sound) and distortion of the EMG signal, indicating that the needle has entered the airway. The needle is advanced until resistance is met, inferring that the posterior plate of the cricoid has been reached. The needle is then further advanced slowly and firmly in a controlled manner through the cartilage, taking care not to breach the mucosa posterior to the PCA muscle. A gentle twisting motion may assist the needle through the cartilage. The return of crisp, sharp EMG potentials indicates the tip of the needle is near the motor end plates of the PCA muscle. The patient is asked to sniff in to confirm the needle position before the predetermined aliquot of BoNT-A is injected. See supplementary material for a video demonstrating this technique.

#### 3.2.3. Cricothyroid (CT) Muscle Injection for ABLD

**Figure 7:**
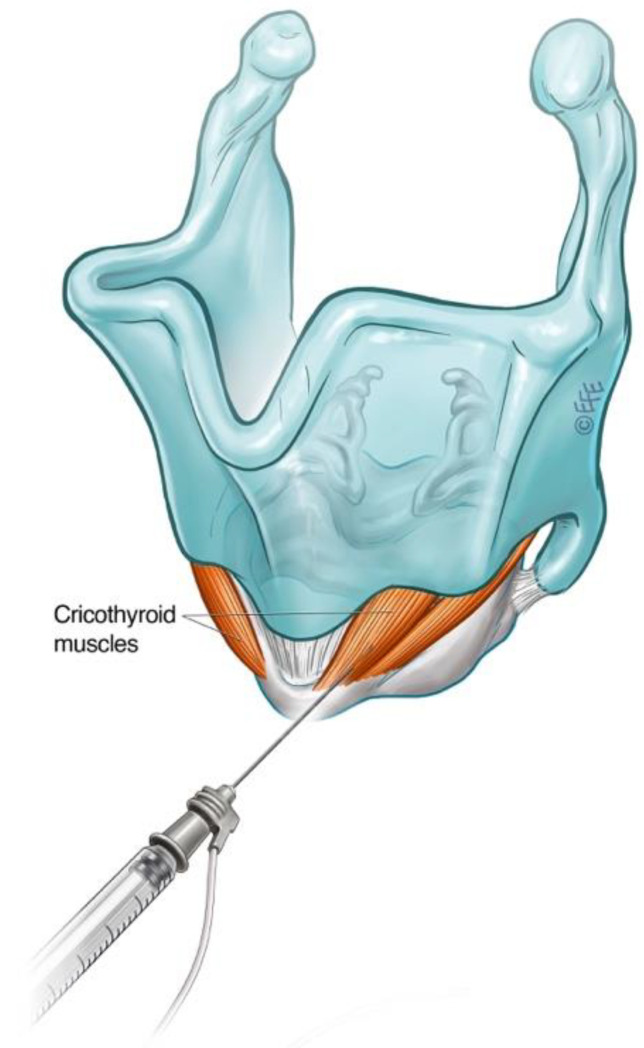
Schematic diagram illustrating technique for injection into the cricothyroid muscle.

Spasmodic bursts of heightened activity in the CT muscle have been observed in some patients with ABLD, with inappropriate CT activity demonstrated on LEMG [103]. This contrasts with other types of laryngeal dystonia, where abnormal CT activity was not found on fine-wire EMG [62,78]. Some 60% of patients in this subgroup reported voice improvement following selective treatment of the CT muscle with BoNT-A [65]. CT muscle injections may therefore be considered in the treatment of ABLD in those with ongoing voice symptoms despite adequate treatment of the PCA or in the presence of a narrow airway precluding further PCA injections. The CT is primarily a tensioner and lengthening of the vocal fold via the cricothyroid joint but also exhibits weak adductor action. The CT muscle is large and readily accessible via an anterior transcutaneous approach (Figure 7).

#### 3.2.4. Dosing and Laterality Considerations for ABLD

BoNT-A dosage for the treatment of ABLD varies widely from 3.75 units to 10 units [16,104,105]. Both bilateral and unilateral injections have been described [16,104]. The authors’ preference is to offer 3.75 units into the more active PCA muscle at initial treatment, followed by a clinical review at 2 weeks post-injection to assess response. The desired dose and concentration of BoNT-A is prepared using a standard stock (2.5 units of Botox^®^ in 0.1 mL of 0.9% NaCl) either directly or diluted with normal saline. For doses greater than 5.0 units, double-strength stock (5.0 units of Botox^®^ in 0.1 mL of 0.9% NaCl) can be prepared to reduce the risk of volume diffusion to the contralateral side or to the inferior constrictor muscle.

Patients with ABLD generally have poorer self-reported voice outcomes than the ADLD group [96]. ABLD can be challenging to manage, and a careful and graduated approach is therefore recommended with a regular clinical assessment to evaluate treatment efficacy.

The technique for BoNT-A treatment delivery to the PCA will depend on the clinician's experience and comfort with each of the described approaches. Patient anatomy is also an important factor, as it can be more difficult to accurately localise the PCA using the lateral rotational approach when the patient has a thick neck [40]. Approximately 25% of patients will respond adequately to the unilateral treatment of PCA [16]. The primary goal is control of breathy voice breaks or complete immobilisation of unilateral PCA function during each treatment cycle [104]. Larger doses can be deployed to achieve this. BoNT-A dose should be adjusted individually for each patient based on an endoscopic assessment of PCA activity and the length of which the treatment cycle lasts. In the authors’ experience, the required dosage of BoNT-A typically varies between 3.75 to 10.0 units for unilateral PCA immobilisation [16,104,105]. The decision of whether to treat the same side at each treatment cycle or whether to alternate sides is reached in discussion with the patient.

Up to 80% of patients with ABLD require treatment beyond the unilateral PCA muscle [16]. The authors recommend unilateral dosing of the more active PCA muscle, as described above. Follow-up laryngoscopy at 2–4 weeks helps discern between under-dosing or technical failure of the treated side and ongoing contralateral abductor pitch breaks. In order to minimise the risk of airway complications, smaller doses of 0.625 units to 2.5 units may be used to weaken but not immobilise the contralateral side [16]. The use of higher staged doses, such as 5.0 units on each side, has also been described [104,105], but the authors recommend proceeding with caution.

Bilateral synchronous treatment of the PCA muscle for ABLD has been reported as safe and effective. The use of a lower dosage regime (1.25 to 1.70 units on one side and 0.9 units on the other side) did not precipitate any breathing difficulties in the patients treated [102]. Stong et al. found a 5% incidence of significant dyspnoea with synchronous BoNT-A injections into PCA muscles with 2.0 to 2.5 units on each side [106]. Improvement in voice with only mild shortness of breath was reported by 89% of patients receiving bilateral synchronous BoNT-A injections to the PCA [107]. Treatment decisions regarding staged or synchronous PCA injections should be informed by clinician experience and extensive discussion with the patient.

### 3.3. Mixed Laryngeal Dystonia (Mixed LD)

Mixed LD is a rare form of laryngeal dystonia presenting with features of both adductor and abductor LD, thought to comprise 1–5% of all presentations of LD [2,59]. Affected individuals experience both abductor and adductor vocal spasms during speech. Diagnosis of this condition is challenging as voice presentation is atypical and may not fall into the usually recognised patterns. In addition, the authors have encountered compensatory functional overlay in their clinical experience. Clinical findings of supraglottic hyperfunction and inhalational speech are common among this group of patients [2]. In comparison to ADLD and ABLD, patients affected by mixed LD struggle with both voice-weighted and voiceless-weighted sentences. Typical features in the clinical history including a positive response to alcohol and non-responsiveness to speech therapy [4,67]. Furthermore, mixed LD patients will have often failed a trial of laryngeal botulinum toxin injection targeted for either ADLD or ABLD. Therefore, a high index of suspicion must be exercised when a patient with suspected LD reports no or limited improvement with adductor or abductor-targeted chemodenervation. Routine evaluation of patients commencing BoNT treatment for LD is recommended by the authors 2–4 weeks after treatment to check treatment responses based on the patient’s self-reported outcome measures in conjunction with endoscopic evaluation and perceptual voice analysis [90]. Patients with mixed LD may present with predominantly adductor or abductor voice pitch breaks, towards which initial treatment should be directed. Perceptual voice strain and tightness after BoNT-A treatment to PCA for a presumed case of ABLD should raise the possibility of mixed dystonia. Breathiness after BoNT-A treatment to the adductor complex for ADLD may be more difficult to evaluate. This symptom itself is an expected initial side-effect following BoNT-A treatment for ADLD. In these cases, voiceless-weighted phrases can help to unmask adductor pitch breaks both perceptually and endoscopically for the astute clinician.

Mixed LD is challenging to treatment [108]. Patients should be counselled for close follow-up over a period of time and may require an individualised dosing regimen optimised for their condition. We find that baseline and serial voice recording after each treatment, along with longitudinal self-reported voice outcome measures, are useful in guiding treatment [90].

Both the adductor and abductor muscles serve as targets for BoNT chemodenervation. The options for the technical approach to each muscle group have been described in previous sections of this article. Unilateral treatment of the PCA and TA complex is initially recommended. As a starting point, a dose of 3.75 units in 0.15 mL for the PCA [16] and 1.0 units in 0.1 mL for the TA complex may be employed [2] (Table 4). The patient may be followed up at 2 weeks for the perceptual and endoscopic assessment to help guide subsequent dosing and injections. Treatment can be prescribed to the contralateral TA complex in the presence of ongoing adductor pitch breaks. In the event of persistent breathiness following treatment, the authors find laryngoscopy with stroboscopy useful in differentiating the cause between adductor paralysis or contralateral abductor muscle hyperfunction [74]. The latter should prompt treatment of the contralateral PCA muscle.

### 3.4. Other Types of Laryngeal Dystonia

#### 3.4.1. Adductor Laryngeal Breathing Dystonia (ALBD)

In addition to affecting the voice, adductor muscle spasms during inspiration can be the predominate feature in a subtype of laryngeal dystonia, producing paradoxical vocal fold motion and stridor. This unusual subtype of LD, in which the primary abnormality is associated with respiration rather than phonation, was observed in the early 1990s [15]. This condition is now widely recognised as adductor laryngeal breathing dystonia (ALBD), sometimes known as respiratory laryngeal dystonia [64,109]. In ALBD, the voice is usually normal, with an absence of the characteristic adductor pitch breaks seen in ADLD. The main clinical features comprise persistent stridor, which may vary from moderate to severe. Affected patients may also complain of a dystonic cough, paroxysmal sneezing or hiccups [63,109]. The respiratory symptoms are often exacerbated by physical exertion. Interestingly, oxygen desaturation is not typically observed despite significant stridor [63,64]. On laryngoscopic examination, paradoxical vocal fold movement can be seen on inspiration, which adductor muscle spasms being triggered by a normal level of respiratory effort, resulting in significant narrowing at the glottic level, leading to stridor. The injection of BoNT-A into TA muscles is effective in treating ALBD. Nine patients with ALBD received 0.625–3.75 units of BoNT-A into each TA muscle in a retrospective case series, depending on the severity of symptoms [63] (Table 4). A statistically significant improvement in function of 55% (range 30–90%) was demonstrated [63]. The adverse effect of transient breathy voice and mild choking on liquids in 5 patients did not persist beyond 2 weeks [63].

A more recent prospective case series conducted by Tierney et al. deployed a wider range of management options. Of 16 patients, 100% underwent respiratory retraining therapy, 68.8% received laryngeal BoNT-A injections, and 31.3% required a tracheostomy for symptomatic relief [64]. The group concluded that although benzodiazepines, anticholinergics, dopamine blockers, neurogenic modulators, tricyclic antidepressants, and anti-reflux medication have been tried in patients with ALBD, all of these have failed to incite an improvement. To date, the BoNT-A injections into the adductor muscle complex remain the most effective treatment. ALBD is a rare but severely disabling condition for which there are limited treatment options, making it very challenging to manage.

#### 3.4.2. Singer’s Dystonia

It is traditionally thought that laryngeal dystonia only affected the task of speaking. LD patients are usually able to sing, laugh and express other emotions vocally without dystonic spasms. A 7-year experience described by Chitkara et al. [60] identified a subgroup of patients with laryngeal dystonia of the singing voice without affected conversational speech at initial presentation. Of the 5 patients in this case series, 80% were females, 80% exhibited adductor pitch breaks when singing, and the mean age of onset was 35.8 years. Of these patients, 60% had received classical training in singing. All genres, including opera, folk, pop, and musical theatre, were involved, with all pitch ranges (top, middle or low) affected. The mainstay of effective treatment was voice therapy used in conjunction with BoNT-A injections into the TA muscle. It was observed that patients with singer’s dystonia exhibited narrower margins of tolerance to the undesirable side-effects of BoNT-A chemodenervation, which included a reduction in volume, decreasing vibrato and truncated pitch range in their singing voice. As such, a smaller average dose of 0.25 units was advocated for use in each TA muscle (Table 4). This rare clinical presentation has since been expanded upon by Halstead et al. [110], who observed that singing pitch breaks were reproducible at specific pitches that are unrelated to the passagio or occurred while performing specific tasks such as singing voiceless consonants. Singer’s dystonia is often misdiagnosed, with the patient’s singing difficulties commonly but incorrectly attributed to problems with technique, including increased muscle tension, register transition or wobble. Nevertheless, it is an important diagnosis to make due to its detrimental ramifications on an individual’s career and psychological ability relative to their ability to perform.

## 4. Adverse Effects and Development of Resistance to BoNT-A

Whilst considered a safe and effective treatment modality for LD, there are some commonly reported side-effects associated with the injection of BoNT-A into the small intrinsic laryngeal muscles. The adverse effects experienced are related to the chemodenervation of the muscle being targeted. In ADLD, where the laryngeal adductors (TA and LCA) are injected, the main adverse effects are that of a weak and breathy voice with a reduced ability to project [2]. In ABLD, the most reported side-effect is dyspnoea due to the immobilisation of the PCA from the toxin [2]. In severe cases, bilateral abductor paralysis may occur, causing respiratory distress. Patients may need to be admitted to the intensive care unit for close monitoring [111]. There have been rare reports of some patients requiring a tracheostomy [100]. In addition, transient dysphagia is another well-reported adverse effect due to the local diffusion of toxins into surrounding tissues [96]. Where side-effects are severe and intolerable, the use of pyridostigmine (a reversible acetylcholinesterase inhibitor) may be considered with reports of significant symptomatic improvement [112].

Rarely, the use of BoNT can be complicated by the development of antibodies which can attenuate or negate the toxin’s therapeutic effects [113]. Factors which have been associated with resistance to BoNT include shorter intervals between doses (booster injections) [114,115], higher doses given per injection cycle [116] and elevated amounts of antigenic protein [113]. Serotype-specific factors such as formulation, manufacturing and storage of toxins may also contribute to the immunogenicity of BoNT. Various structural and bioassays are available to detect BoNT antibodies, but they are generally expensive, difficult to access and require sacrificing animals. Hence clinical tests are increasingly used to detect immunoresistance. These tests have the advantages of being easy to administer, simple to interpret, as well as exhibiting reliable clinical correlation. The ‘frontalis anitbody test’ [117] and the ‘unilateral brow injection test’ [118] are frequently used to evaluate a patient’s sensitivity to BoNT. A low dose of BoNT is injected unilaterally into the frontalis or corrugator/procerus muscles. The resting and frowning facial expressions of the patient are then assessed at 1–3 weeks. If asymmetry is observed, then that would imply the patient remains sensitive to BoNT, suggesting the absence of neutralising antibodies.

## 5. Assessment of Treatment Outcomes

The objective of any given intervention is to eliminate clinical symptoms and to the patient’s quality of life. There is no universally accepted specific battery of objective tests for use to measure treatment outcomes from BoNT-A in LD. Currently, many researchers rely on patient-reported outcome measures (PROMS) such as Voice Handicap Index (VHI), Percentage Normal Function (PNF) scores, and Voice-Related Quality Of Life (V-RQOL) to assess treatment outcomes. It is important to note that none of these questionnaires is specifically designed to assess LD severity or symptomatology.

Lundy found significant correlation between voice quality and the severity of vocal symptoms prior to BoNT-A treatment [119]. The length of treatment response was greater in male patients [120]. Wingate used the Voice Handicap Index (VHI) and Social Readjustment Rating Scale (SRRS) to assess patient perception pre- and post- BoNT-A in a cohort of over 65 year olds [121]. The results indicated no significant correlation between VHI scores, voice severity or SSRS ratings. Morzaria et al. also noted that there is no consensus on which QOL instrument should be used in assessing treatment outcomes in LD [122]. The group found that VHI, VHI-10 and V-RQOL were highly correlated in subscale and total scores in a study involving 37 patients. All 3 scores were significantly responsive to BoNT-A therapy [122]. Using VHI-10 and PNF, Simpson et al. showed significant improvement in both measures in a cohort of patients who received supraglottic BoNT-A treatment for ADLD [76]. Paniello’s prospective, non-randomised case series collected V-RQOL scores at 4-week intervals over 22 patient treatment cycles. It found that although QOL had improved for ADLD patients undergoing BoNT-A treatment, they still spend a significant proportion of each treatment cycle with a reduced QOL [123]. Shoffel-Havakuk et al. [124] established the validity of the OMNI Vocal Effort Scale (OMNI-VES) for rating perceived voice-rating perception in patients with ADLD and concluded that it could be used to evaluate response for BoNT-A injection treatment. Interestingly, only a weak correlation was found between the OMNI-VES and the more widely circulated Voice-Related Quality of Life (V-RQOL) scores in a case group of 178 patients. No significant correlation was found between the OMNI-VES and the clinician-completed Consensus Auditory-Perceptual Evaluation of Voice (CAPE-V).

Novakovic et al. studied the longitudinal effect of BoNT-A treatments for ADLD on functional outcomes and quality of life. A mean improvement of 9.6 and 30.3% was noted in VHI and PNF scores, respectively, across 1457 injection treatments in 133 patients over 36 months. There was a significant correlation between the VHI-10 and PNF scales [90]. Rubin et al. also studied the longitudinal effect of BoNT-A treatment but looked specifically at the V-RQOL outcome measure. Statistically significant improvements in mean total and domain V-RQOL were found in 42 patients over a 38-month period [125].

A meta-analysis assessed the efficacy of BoNT-A on the treatment of LD by engaging in a ‘best synthesis’ systematic summary, where 97% improvement was found as a result of BoNT-A treatment in a review of 22 studies [126]. Faham’s systemic review and meta-analysis assessing the quality of life (QOL) after botulinum toxin injections in ADLD patients with data from 9 studies also concluded that BoNT-A injections had a positive effect on patient QOL [127]. A Cochrane review from 2006 only identified one study in meeting the inclusive criteria, which reported a treatment vs no treatment comparison [128].

The challenge to find simple yet reproducible outcome measures that are capable of tracking longitudinal treatment outcomes over time across multiple domains is highlighted by a recent comprehensive systemic review by Rumbach et al., who investigated treatment outcome measures for LD. The review concluded that there is currently no unified approach to the measurement of outcomes in LD treatment research. It is recommended that a core outcome set be developed and implemented to facilitate the assessment of current and new treatments for LD [129].

## 6. Discussion and Future Perspectives

Multifactorial in its etiology, LD is a group of phenotypically complex and heterogeneous disorders that requires a multidisciplinary approach for effective management. Its diagnosis is based on a clinical approach which is open to bias. Consensus between clinicians is difficult to achieve. A recent multidisciplinary expert update on LD research has concluded that the highest priority for the future is the clinical implementation of objective, disease-specific and pathophysiologically relevant biomarkers. These need to be fast, accurate and cost-effective in diagnosing LD and differentiating it from other similar conditions [4].

Various surgical treatments have been reported for LD in the literature, including recurrent laryngeal nerve section [130], selective laryngeal adductor denervation-reinnervation (SLAD-R) [131], Type II midline lateralisation thyroplasty [132,133], thyroarytenoid myoneurectomy [134] and more recently, radiofrequency-induced thermotherapy [135], none of which have been successful at achieving long-term symptom control. Botulinum neurotoxin chemodenervation prevails as the standard of care in LD, with a large body of evidence attesting to its efficacy [2,59,128,136]. In Japan, where BoNT-A injections have historically been utilised in an off-label capacity for the treatment of LD, Hirose et al. have been able to demonstrate its therapeutic efficacy through a placebo-controlled, randomised, double-blind clinical trial [95]. Along with the work of Hyodo et al. [5,137], BoNT-A therapy has finally been accepted and funded by the Japanese medical insurance scheme as a treatment for LD.

The alcohol-responsive nature of LD formed the basis of investigation for a novel pharmacological agent. The sodium salt of gamma-hydroxybutyric acid (GHB) and sodium oxybate (Xyrem^®^) mimics some of the effects of alcohol. In an open-label study of sodium oxybate in 25 patients with 45 patients with LD, voice symptoms were reduced in 82.2% of patients with an alcohol-responsive form of LD [138]. Results from a new randomised, placebo-controlled, double-blind clinical trial [NCT03292458] are eagerly anticipated.

As we continue to gain a better understanding of the role phenomenology, genetics, and central nervous system (CNS) abnormalities play in the pathophysiology of LD, promising new and novel therapy approaches are being trialled. Some target the CNS, whilst others focus on the larynx. Deep brain stimulation (DBS) has been established as an effective treatment for severe movement disorders over the past 30 years. Its role in the treatment of LD is being investigated [139]. A Phase 1 prospective, randomised, double-blind, crossover trial has shown promising results, confirming the safety of DBS in LD patients [140]. The Thalamic Deep Brain Stimulation for Spasmodic Dysphonia (DEBUSSY) clinical trial [NCT03292458] has recently been completed, pending report. If findings show sufficient safety and efficacy, it may pave the way for DBS to be introduced as an accepted treatment option in LD.

Targeting the somatosensory dysfunction component of LD pathophysiology, a recent study showed significant improvement in symptoms in 69% of patients when the one-time, 40-min application of non-invasive laryngeal vibrotactile stimulation (VTS) was applied [141]. Positive changes in the somatosensory region of the motor cortex were demonstrated, along with a carryover effect of at least 20 min duration after VTS was discontinued [141]. A new clinical trial is underway to delineate the therapeutic dosage of VTS therapy for effective vocal improvement in LD [NCT03746509].

## 7. Conclusions

Laryngeal dystonia is a rare condition with various clinical phenotypes, most commonly affecting voice function. The underlying pathophysiology is complex with structural and functional components, our understanding of which continues to evolve. BoNT injection of the end organ provides temporary symptom relief in LD, with attendance required roughly every 3 months for repeat treatment, which can be both psychologically and financially burdensome over a lifetime [142]. Despite its shortcomings, BoNT is the most effective and reliable treatment modality for LD at the present time and remains the current standard of care. In order to optimise outcomes in LD patients, it is important to assess, evaluate and adjust the dose of BoNT at each treatment cycle as necessary, based on the patient’s response, including monitoring of side effects and longitudinal voice function outcomes. Where dose adjustment provides an unsatisfactory balance between side effects and improved function, alternative dosing regimens and approaches can be employed. This article aims to provide a comprehensive overview of the clinical application of botulinum neurotoxin in the treatment of laryngeal dystonia, with descriptions of the full arsenal of injection techniques and approaches to enable the astute clinician to manage this condition effectively.

## Figures and Tables

**Table 1:** Classification of laryngeal dystonia, muscle groups affected and main clinical features [2,57,60,61,62,63,64].

Laryngeal Dystonia Type	Muscle Group(s) Predominantly Affected	Main Clinical Features
Adductor laryngeal dystonia (~80%)	Adductors (Predominantly TA, but can also affect LCA, IA and CT)	Tight, strained voice with characteristic adductor pitch breaks
Abductor laryngeal dystonia (~15%)	Abductors (PCA)	Breathy, asthenic voice with characteristic abductor pitch breaks
Mixed laryngeal dystonia (~5%)	Adductors and abductors	Features of both ADLD and ABLD, challenging to diagnose and manage
Adductor respiratory laryngeal dystonia (Uncommon)	Adductors	Persistent stridor due to paradoxical vocal fold motion from abnormal inspiratory adductor activity, speech unaffected
Singer’s dystonia (Rare)	Adductors, when singing	Adductor pitch breaks on singing only, speech unaffected

**Table 2:** Examples of sentences in the English language featuring voice-weighted (vowels), helpful in eliciting adductor pitch breaks in ADLD.

ADLD: Voice-Weight Sentences (Vowel Sounds: a, e, i, o, u)
‘We eat eels every Easter.’
‘Tom wants to be in the army.’ ‘We mow our lawn all year.’ ‘I hurt my arm on the iron bar.’ ‘Ada and Eve ate oysters at the oyster bar.’

**Table 3:** Examples of sentences in the English language featuring voiceless-weighted (consonants) sentences, helpful in eliciting breathy pitch breaks in ABLD.

ABLD: Voice-Less Weighted Sentences (Consonant Sounds: h, s, p, t, k)
‘Harry hung his hat on the hook.’
‘Cake and ice-cream are tasty treats.’ ‘Patty helped Kathy carve the turkey.’ ‘A mahogany highboy isn’t heavy.’ ‘Potato soup tastes fine with crackers.’

**Table 4:** Suggested initial onabotulinum toxin A (Botox^®^) dosing regimen for laryngeal dystonia [2,16,60,63,64,65,75,104].

Laryngeal Dystonia Type	Target Muscle	Suggested Botox^®^ Dosing (Each Side)—Bilateral Treatment. In Units	Suggested Botox^®^ Dosing—Unilateral Treatment. In Units (U)
Adductor laryngeal dystonia	TA/LCA	0.6–1.3	2.5–3.75
	Supraglottis	7.5	-
	IA	-	2
Abductor laryngeal dystonia	PCA	1.25–2.5	3.75–10
	CT	3.75–5	-
Adductor breathing laryngeal dystonia	TA/LCA	0.625–3.75	2.5–5
Singer’s dystonia	TA	0.25–0.5	0.5–1.0

## Data Availability

Not applicable.

## Supplementary Materials

The following supporting information can be downloaded at: https://www.mdpi.com/article/10.3390/toxins14120844/s1, Video S1: Trans-airway injection approach to the PCA muscle for ABLD.

## Abbreviations

BoNT	Botulinum neurotoxin
ABLD	Abductor laryngeal dystonia
ADLD	Adductor laryngeal dystonia
ALBD	Adductor laryngeal breathing dystonia
LD	Laryngeal dystonia
RLN	Recurrent laryngeal nerve
SLN	Superior laryngeal nerve
LEMG	Laryngeal electromyography
CNS	Central nervous system
